# Establishment, FEM analysis and experimental validation of tooth movement prediction model of orthodontic archwire T-loop

**DOI:** 10.1186/s12903-022-02430-9

**Published:** 2022-09-17

**Authors:** Jingang Jiang, Liang Yao, Yongde Zhang, Xuefeng Ma, Yafeng Guo, Yi Liu

**Affiliations:** 1 Key Laboratory of Advanced Manufacturing and Intelligent Technology, Ministry of Education, Harbin University of Science and Technology, Harbin, 150080 Heilongjiang People’s Republic of China; 2 Robotics & Its Engineering Research Center, Harbin University of Science and Technology, No. 52, Xuefu Road, Nangang Dist, Harbin, China; 3 State Key Laboratory of Robotics and System, Harbin Institute of Technology, Harbin, 150001 Heilongjiang People’s Republic of China; 4 Peking University School of Stomatology, Beijing, 100081 China

**Keywords:** Tooth movement prediction model, T-loop, Finite elements method (FEM) analysis, Orthodontic force measurement

## Abstract

**Background:**

The T-loop has been used clinically to close gap between teeth. And it is a typical orthodontic archwire bending method. However, the design of the T-loop parameters for different patients is based on the clinical experience of the dentists. The variation in dentists' clinical experience is the main reason for inadequate orthodontic treatment, even high incidence of postoperative complications.

**Methods:**

Firstly, the tooth movement prediction model is established based on the analysis of the T-loop structure and the waxy model dynamic resistance. As well as the reverse reconstruction of the complete maxillary 3D model based on the patient CBCT images, the oral biomechanical FEM analysis is completed. A maxillary waxy dental model is manufactured to realize the water-bath measurement experiment in vitro mimicking the oral bio-environment. Thus, the calculated, simulation and experimental data are obtained, as well as obtaining a cloud of total deformation from the simulation analysis.

**Results:**

The growth trend of the 11 sets of simulation data is the same as that of the experimental data. And all of them show that the tooth displacement is positively correlated with the cross-sectional size of the archwire, and the clearance distance. As well as the higher Young's modulus of the archwire material, the greater the tooth displacement. And the effect of archwire parameters on tooth displacement derived from simulation and experimental data is consistent with the prediction model. The experimental and calculated data are also compared and analyzed, and the two kinds of data are basically consistent in terms of growth trends and fluctuations, with deviation rates ranging from 2.17  to  10.00%.

**Conclusions:**

This study shows that the accuracy and reliability of the tooth movement prediction model can be verified through the comparative analysis and deviation calculation of the obtained calculated, simulation and experimental data, which can assist dentists to safely and efficiently perform orthodontic treatment on patients. And the FEM analysis can achieve predictability of orthodontic treatment results.

## Background

In recent years, oral malocclusion has shown a high prevalence and is one of the three major oral diseases [1]. It affects oral function, craniofacial development, appearance and interpersonal relationships, and even causes psychological disorders in minors [2, 3, 4]. Orthodontic techniques can effectively treat malocclusion. During orthodontic treatment, due to the phenomenon of orthodontic crowding, some teeth are selectively extracted and a corresponding extraction gap is formed. For this reason, the closure of the extraction gap is a very important aspect [5], which is directly related to the smooth implementation of the entire orthodontic plan [6, 7, 8].

T-loop is widely used to close gap between teeth due to the structural characteristics that can generate horizontal, vertical and torsional force [9, 10, 11, 12, 13]. During the process of closing gap, the teeth are subjected to both the orthodontic force generated by the T-loop and the biological resistance of the periodontal tissues [14, 15, 16]. In turn, the orthodontic force generated by the archwire are inextricably linked to the clearance distance, cross-sectional size and material properties of the T-loop [17, 18].

In traditional orthodontic diagnosis, depending on the treatment stage and specific orthodontic purpose, dentists select archwire parameters empirically [19, 20, 21, 22]. This leads to unpredictable tooth movement, and can easily cause irreparable damage to the patients and reduce treatment efficiency [13]. During the treatment, if the tooth movement is too small, the orthodontic effect is not obvious, and if the tooth movement is too large, it can increase the patient's dental pain, leading to periodontal tissue damage, tooth loosening, and even serious root and alveolar bone resorption problems [23, 24, 25]. Therefore, dentists will find the optimal position by repeatedly adjusting the parameters of the orthodontic archwire. However, repeated adjustments can reduce the mechanical properties of the orthodontic archwire. And it also increases the clinical treatment time and causes inconvenience to both the dentist and the patient. Therefore, by revealing the relationship between archwire parameters, orthodontic force and tooth movement, it enables the dentist to develop an individualized orthodontic treatment plan by taking into account the patient's malocclusion, the mechanical behavior of archwire and the tooth movement pattern. This further improves the efficiency and safety of fixed orthodontic treatment and reduces the patient's pain.

In this paper, based on the dynamic resistance model of the waxy model and the dynamic orthodontic force model of the T-loop, a tooth movement prediction model is established to realize the tooth movement prediction analysis. The T-loop parameters and tooth movement distance are quantified. And FEM analysis and experimental validation are both conducted. Experimental data are collected using the designed tooth movement measurement method. And the accuracy of the tooth movement prediction model is demonstrated by data comparison and deviation analysis.

Therefore, the tooth movement prediction model established in this paper can assist dentists in selecting the appropriate T-loop parameters for the movement distance and angle of the tooth. Thus, it provides a theoretical basis for the development of individualized orthodontic treatment plans for patients. At the same time, it reduces the clinical treatment time of patients and dentists, improves orthodontic efficiency, as well as achieves predictability of orthodontic treatment effect.

## Methods

### Dynamic resistance analysis of waxy model

**Fig. 1:**
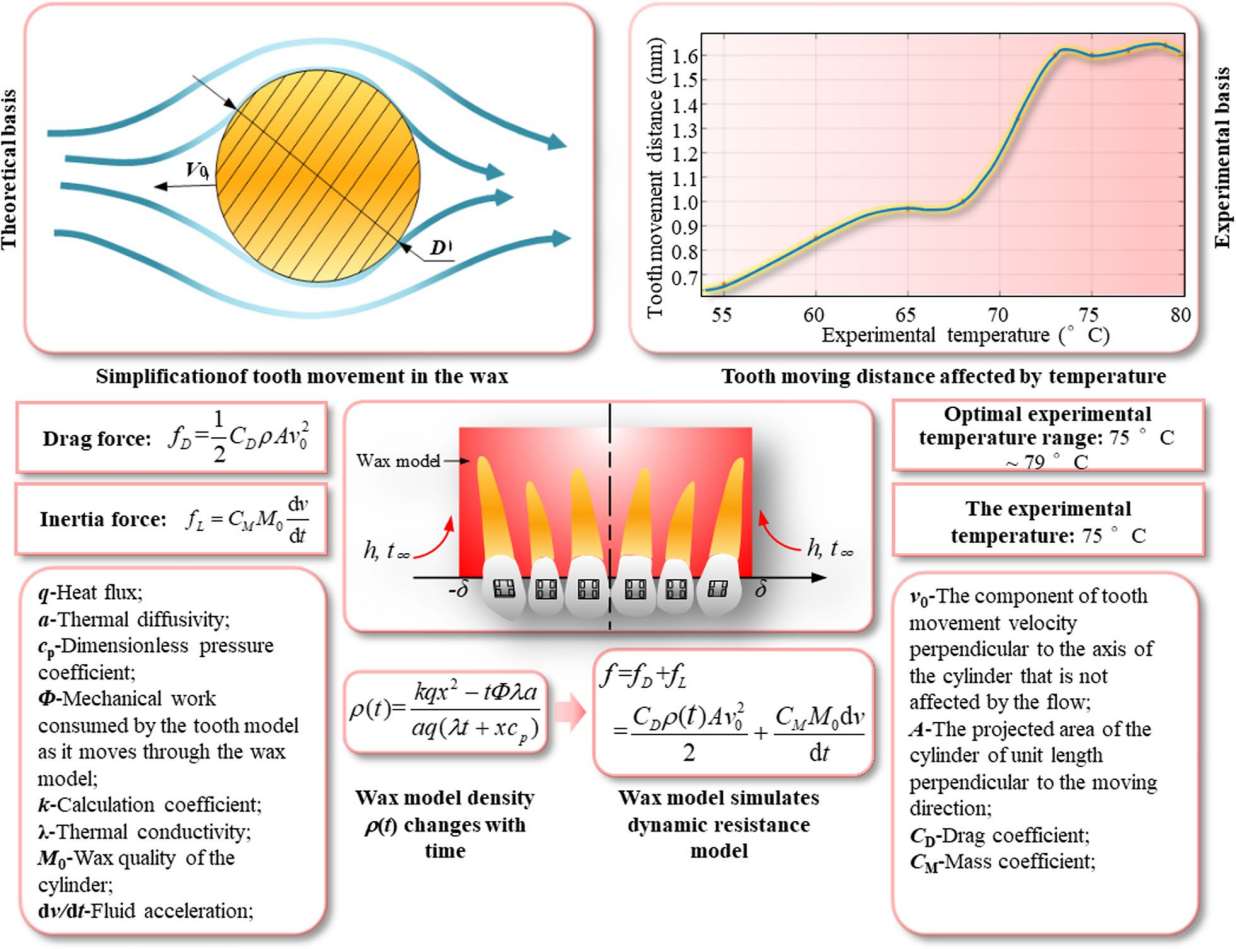
Waxy dynamic resistance model during tooth movement

In order to restore the relative tooth movement under realistic conditions, a non-rigid waxy model is chosen to simulate the biological properties of the periodontal environment. Non-rigid waxy models are widely used in dental clinical experiments and training because the viscous fluid properties vary with temperature, such as the Typodon orthodontic training model [26, 27]. As well as Liu et al. experimentally verified the feasibility of the waxy model for simulating tooth movement at a certain temperature [28]. Meanwhile, according to the theory of viscous fluid dynamics, during orthodontic treatment, the impedance force of tooth in waxy model is composed of resistance and inertia force [29]. Through experimental verification, the optimal water-bath temperature for tooth movement in the waxy model is determined to be 75 °C [30, 31]. At the same time, an expression for the variation of the waxy dental density with time is established. According to the expression, the resistance equation and the inertia force equation, the dynamic resistance model of the waxy dental model in the process of simulated tooth movement is established [32, 33], as shown in Fig. 1.

### Modeling of dynamic orthodontic force in T-loop

During orthodontic treatment, the tooth movement is influenced by a combination of the periodontal tissue's biological resistance and the orthodontic force generated by the archwire. Besides, orthodontic force changes dynamically as treatment progresses [32]. Therefore, before establishing a tooth movement prediction model, a dynamic orthodontic force model needs to be established.

**Fig. 2:**
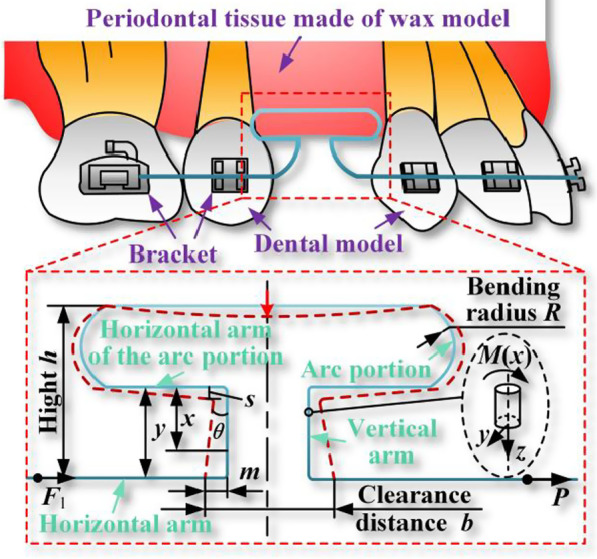
T-loop deformation and vertical arm force analysis

Analysis of T-loop working as follows: the dentist pulls the horizontal arm of T-loop to create a clearance, causes elastic deformation of the vertical arm and the arc portion, and then fixes the horizontal arm on the bracket. The tooth is pulled to a predetermined position using the restoring force of the T-loop itself, thus completing the closure of the gap between teeth [19, 20]. At the same time, T-loop with symmetrical structure is used, that is, the Alpha/Beta structure on both sides of T-loop is equal, so that the orthodontic force released by the horizontal arm can be equal and opposite [34]. Therefore, only one side of T-loop needs to be analyzed. The T-loop deformation analysis is shown in Fig. 2.

The orthodontic force generated by the T-loop is composed of the vertical arm and the arc portion of the deformation superimposed. So the vertical arm and the arc portion are modeled mechanically separately. And then the total orthodontic force of the T-loop is calculated by superposition. Firstly, the vertical arm is subjected to force analysis and mechanical modeling. As shown in Fig. 2, the bending radius of the arc portion is *R*, the total height is *h*, the length of the vertical arm is *y*. By pulling the horizontal arm of the T-loop creates a clearance distance *b*. And the component of the force applied to the horizontal arm that deforms the vertical arm is *p*.

The approximate differential equation for the vertical arm deflection curve is as follows:

1 $$\frac{{{\text{d}}^{2} v}}{{{\text{d}}x^{2} }} = \frac{M(x)}{{EI_{z} }}$$

where *E* is the elastic modulus of material, *M*(*x*) is the bending moment of the vertical arm at *x* distance. *I*_*z*_ is the rotational inertia of the archwire *z*-axis. The rotational inertia of a round archwire is *I*_*z*_ = *πD*^4^/64, and *D* is the diameter of the round archwire. For a rectangular archwire *I*_*z*_ = *c*_1_*c*_2_^3^/12. The *c*_1_ is length of the side perpendicular to the z-axis on the rectangular archwire cross-section. The rotation angle equation *θ*(*x*) and the deflection equation *ν*(*x*) for the vertical arm can be obtained by integrating Eq. (1) once and twice, respectively.

2 $$\left\{ \begin{gathered} \theta (x) = \frac{{{\text{d}}v(x)}}{{{\text{d}}x}} = \int {\frac{M(x)}{{EI_{{\text{z}}} }}{\text{d}}x + C_{0} } \hfill \\ v(x) = \int {\int {\frac{M(x)}{{EI_{{\text{z}}} }}{\text{d}}x{\text{d}}x + C_{0} x + D_{0} } } \hfill \\ \end{gathered} \right.$$

where *C*_0_ and *D*_0_ are integration constants that can be determined by the boundary conditions at special points. The expression for the bending moment of the vertical arm is:

3 $$M(x) = - P(y - x)$$

Substituting Eqs. (1) and (3) into Eq. (2) for integration, obtaining:

4 $$\left\{ \begin{gathered} \theta (x) = \int {\frac{P}{{EI_{z} }}(x - y){\text{d}}x = } \frac{P}{{EI_{z} }}(\frac{{x^{2} }}{2} + yx) + C_{0} \hfill \\ v(x) = \int {\int {\frac{P}{{EI_{z} }}(x - y){\text{d}}x{\text{d}}x = } \frac{P}{{EI_{z} }}(\frac{{x^{3} }}{6} + \frac{{yx^{2} }}{2}) + C_{0} x + D_{0} } \hfill \\ \end{gathered} \right.$$

**Fig. 3:**
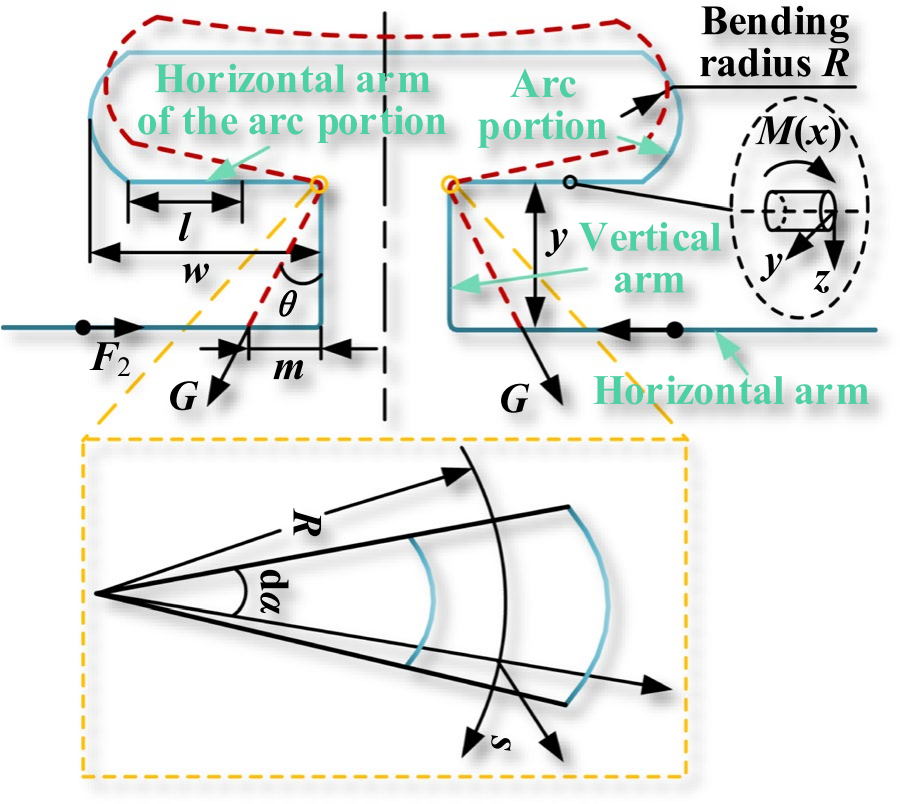
Deformation analysis of nodes and horizontal arm of the arc portion

Using the special points with known deflection and rotation angle, the integration constants in Eq. (4) can be determined. Since the longitudinal symmetry plane exists at the intersection of horizontal arm of the arc portion and vertical arm, and the symmetry plane is subject to external force, the axis of the deformed curved beam is still located within the longitudinal symmetry plane. The intersection point deformation belongs to the plane bending deformation of the curved beam. Therefore, the arc at the intersection can be equated to a curved beam with radius, curvature and section diameter. To take the radius micro-element d*α* cross section, as shown in Fig. 3.

After deformation, the differential equation of the deflection curve at the intersection of horizontal arm of the arc portion and vertical arm is as follows:

5 $$\frac{{{\text{d}}^{2} u}}{{{\text{d}}s^{2} }} + \frac{u}{{R^{2} }} = - \frac{{M_{0} }}{{EI_{\omega } }}$$

where *u* is the displacement of the curved beam in the *x*-direction intersection cross section. The bending moment condition at the intersection is known to be $M_{0} = \left. M \right|_{x = 0} = - Py$. $I_{\omega }$ is the rotational inertia of the curved beam's cross section on the *ω* axis. The vertical arm is consistent with the bending form of the curved beam at the intersection, so $I_{\omega } = I_{z}$. Also it is defined the arc length equation d*s* = *R*d*α*, and obtained the differential equation of the deflection curve at the intersection point after substitution, as shown in Eq. (6):

6 $$\frac{{{\text{d}}^{2} u}}{{{\text{d}}\alpha^{2} }} + u = - \frac{{M_{0} R^{2} }}{{EI_{\omega } }}$$

The non-associative differential equation with constant coefficients for the deflection curve after deformation of the curved beam at the intersection point is:

7 $$u(\alpha ) = A_{0} \cos \alpha + B_{0} \sin \alpha - \frac{{M_{0} R^{2} }}{{EI_{\omega } }}$$

The boundary condition at the intersection is $u{|}_{{\alpha { = }\pi /2}} = 0$, $\left. {\frac{{{\text{d}}u}}{{{\text{d}}\alpha }}} \right|_{\alpha = \pi /2} = 0$. Therefore, it can be determined that *A*_0_ = 0, $B_{0} { = }\frac{{M_{0} R^{2} }}{{EI_{\omega } }}$, and bring them into Eq. (7) to obtain Eq. (8):

8 $$u(\alpha ) = \frac{{M_{0} R^{2} }}{{EI_{\omega } }}\sin \alpha - \frac{{M_{0} R^{2} }}{{EI_{\omega } }}$$

Also, the corner equation of the curved beam can be obtained as:

9 $$\beta (\alpha ) = \frac{{{\text{d}}u}}{{{\text{d}}s}} = \frac{{M_{0} R\cos \alpha }}{{EI_{\omega } }}$$

By introducing of boundary conditions $\left. v \right|_{x = 0} = \left. u \right|_{\alpha = 0} = - \frac{{M_{0} R^{2} }}{{EI_{\omega } }}$, $\left. \theta \right|_{x = 0} = \left. \beta \right|_{\alpha = 0} = \frac{{M_{0} R^{{}} }}{{EI_{\omega } }}$, to obtain $C_{0} = \frac{{M_{0} R^{{}} }}{{EI_{\omega } }}$, $D_{0} = - \frac{{M_{0} R^{2} }}{{EI_{\omega } }}$.

Bringing $C_{0}$ and $D_{0}$ into Eqs. (4) and (10), obtaining:

10 $$\left\{ \begin{gathered} \theta (x) = \frac{P}{{EI_{z} }}(\frac{{x^{2} }}{2} + yx) + \frac{{M_{0} R}}{{EI_{w} }} \hfill \\ v(x) = \frac{P}{{EI_{z} }}(\frac{{x^{3} }}{6} + \frac{{yx^{2} }}{2}) + \frac{{M_{0} R}}{{EI_{w} }}x + \frac{{M_{0} R}}{{EI_{w} }} \hfill \\ \end{gathered} \right.$$

According to the established deflection differential equation, the corresponding boundary conditions are replaced: the maximum deflection value *m*, and the maximum rotation angle at *x* = *y*. The external force that deforms the vertical arm is then obtained as:

11 $$p = - \frac{{3[mEI_{z} + M_{0} R(y - R)]}}{{y^{3} }}$$

Further according to the principle of action and reaction force, the force *P* that deforms the vertical arm is equal to the counter force of the orthodontic force *F*_1_ applied to the tooth during the orthodontic procedure. Thus, it can be obtained that:

12 $$F_{1} = - p = \frac{{3[mEI_{z} + M_{0} R(y - R)]}}{{y^{3} }}$$

As mentioned earlier, the arc portion and the deformation of the vertical arm together constitute the orthodontic force generated by the T-loop during correction. After the analysis of the vertical arm, a mechanical modeling.

analysis of the arc portion is performed. Similar to the analysis of the vertical arm, the horizontal arm of the arc portion as the main deformation part, need to establish the corresponding deformation equation, as shown in Fig. 3.

During the deformation of the T-loop, the position of the connection between the horizontal arm of the arc portion and the vertical arm is constantly changing. It is not easy to measure the distance that the horizontal arm of the arc portion moves. Therefore, it is necessary to simplify the movement of the intersection symmetry center during the T-loop deformation. The symmetry center of the intersection before deformation is overlapped, and the difference between the theoretical length and the initial length of the vertical arm after deformation is calculated. This difference is the bending deformation *s* of the horizontal arm of the arc portion, which can be obtained from the following equation:

13 $$s = \sqrt {m^{2} + y^{2} } - y$$

where *y* is the length of the vertical arm in the natural state and also the bending deflection of the vertical arm. Similar to Eq. (1), the deflection curve differential equation for the horizontal arm of the arc portion is:

14 $$\frac{{{\text{d}}^{2} v}}{{{\text{d}}l^{2} }}{ = }\frac{M(l)}{{EI_{z} }}$$

The corner Eq. (15) and the deflection Eq. (16) for the horizontal arm of the arc portion can be determined by integrating Eq. (14) once and twice, respectively:

15 $$\theta (l) = \frac{{{\text{d}}v(l)}}{{{\text{d}}l}} = \int {\frac{M(l)}{{EI_{{\text{z}}} }}{\text{d}}l + C_{1} }$$

16 $$v(l) = \frac{{{\text{d}}^{2} v(l)}}{{{\text{d}}l^{2} }}{ = }\int {\int {\frac{M(l)}{{EI_{{\text{z}}} }}{\text{d}}l{\text{d}}l + C_{1} } } l + D_{1}$$

where *C*_1_ and *D*_1_ are integration constants, and the bending moment expression for the horizontal arm of the arc portion is:

17 $$M(l) = - G(w - R - l)$$

where *G* is the component that deforms the horizontal arm of the arc portion, *w* is the total length of the horizontal arm of the arc section, and *R* is the bending radius of the arc portion. Substituting Eqs. (14) and (17) into Eqs. (15) and (16) for integration, the following equation is obtained:

18 $$\theta (l) = \int {\frac{G}{{EI_{z} }}(l + R - w)dl = } \frac{G}{{EI_{z} }}(\frac{{l^{2} }}{2} + Rl - wl) + C_{1}$$

19 $$\begin{gathered} v(l) = \int {\int {\frac{G}{{EI_{z} }}(l + R - w){\text{d}}l{\text{d}}l} } \hfill \\ { = }\frac{G}{{EI_{z} }}(\frac{{l^{3} }}{6} + \frac{{Rl^{2} - wl^{2} }}{2}) + C_{1} l + D_{1} \hfill \\ \end{gathered}$$

The lateral arc on one side can be equated to a curved beam of radian *π*/4, and the radian d*β* is taken to be infinitesimal. This is the same process as solving for the vertical arm boundary condition, so the boundary condition can be given as:

20 $$u(\beta ) = A_{1} \cos \beta + B_{1} \sin \beta - \frac{{M_{0} R^{2} }}{{EI_{\omega } }}$$

The boundary conditions of the arc portion are $\left. u \right|_{\beta = \pi } = 0$, $\left. {\frac{{{\text{d}}u}}{{{\text{d}}\beta }}} \right|_{\beta = \pi } = 0$, and the general solutions obtained are $A_{1} = \frac{{M_{0} R^{2} }}{{EI_{\omega } }}$, $B_{1} = 0$. Then the deflection equation of the arc portion is:

21 $$u(\beta ) = \frac{{M_{0} R^{2} }}{{EI_{\omega } }}\cos \beta - \frac{{M_{0} R^{2} }}{{EI_{\omega } }}$$

The corner equation of the arc portion is:

22 $$\varepsilon (\beta ) = \frac{{{\text{d}}u}}{{{\text{d}}s}} = - \frac{{M_{0} R\sin \beta }}{{EI_{\omega } }}$$

Because the boundary conditions are $\left. v \right|_{{l{ = 0}}} = \left. u \right|_{\beta = 0} = 0$ and $\left. \theta \right|_{l = 0} = \left. \varepsilon \right|_{\beta = 0} = 0$, it is able to obtain *C*_1_ = 0 and *D*_1_ = 0. Substituting *C*_1_ = 0 and *D*_1_ = 0 into Eqs. (18) and (19) obtains Eqs. (23) and (24):

23 $$\theta (l) = \frac{G}{{EI_{z} }}(\frac{{l^{2} }}{2} + Rl - wl)$$

24 $$v(l) = \frac{G}{{EI_{z} }}(\frac{{l^{3} }}{6} + \frac{{Rl^{2} - wl^{2} }}{2})$$

The corresponding boundary conditions are substituted as follows: when *l* = *w*-*R*, the horizontal arm of the arc portion reaches the maximum deflection at its end, which is *v* = *s*, approximated by the same Eq. (13). Further can get the bending force of the horizontal arm of the arc portion, as follows:

25 $$G = - \frac{{3sE_{z} }}{{(R - w)^{3} }}$$

From further mechanical analysis, it is obtained that the component force along the horizontal arm should be equal to the orthodontic force *F*_2_ released by the arc portion. The following equation is obtained:

26 $$F_{2} = - G \times \sin \theta = \frac{{3mEI_{z} (\sqrt {y^{2} + m^{2} } - y)}}{{(w - R)^{3} \sqrt {y^{2} + m^{2} } }}$$

From the mechanical analysis, the restoring force from the deformation of the vertical arm and the arc portion together constitute the orthodontic force *F*_0_ released by the horizontal arm. That is, the following equation:

27 $$\begin{gathered} F_{0} = F_{1} { + }F_{2} \hfill \\ { = }\frac{{3[mEI_{Z} + M_{0} R(y - R)]}}{{(y - R)^{3} }} + \frac{{3mEI_{z} (\sqrt {y^{2} + m^{2} } - y)}}{{(w - R)^{3} \sqrt {y^{2} + m^{2} } }} \hfill \\ \end{gathered}$$

### Establishment of tooth movement prediction model

After the previous calculations, the dynamic resistance model for the tooth movement simulated in the waxy model can be expressed by Eq. (28):

28 $$f = f_{D} + f_{L} = \frac{1}{2}C_{D} \rho (t)Av_{0}^{2} + C_{M} M_{0} \frac{dv}{{dt}}$$

where *f* is the dynamic resistance to tooth movement in the waxy model. Bringing in the orthodontic force *F*_0_ released by the T-loop, it is obtained that:

29 $$\begin{gathered} F{ = }F_{0} - f{ = }F_{0} - f_{D} - f_{L} \hfill \\ { = }F_{0} - \frac{1}{2}C_{D} \rho (t)Av_{0}^{2} - C_{M} M_{0} \frac{{{\text{d}}v}}{{{\text{d}}t}} \hfill \\ \end{gathered}$$

In physics, the relationship between the displacement *r* and the velocity vector *v*, Eq. (30), can be determined by integrating:

30 $$\left\{ \begin{gathered} r = \, r_{0} + \int {vdt} \hfill \\ v = \, v_{0} + \int {a{}_{0}dt} \hfill \\ \end{gathered} \right.$$

In Newton's Second Law, the relationship between the acceleration *a*_0_, the mass *m*_0_ and the force *F* can be expressed by Eq. (32):

31 $$F{ = }m_{0} a_{0}$$

The acceleration *a*_0_ of the tooth moving in the waxy model, subject to orthodontic force *F*_0_, inertial force *f*_L_ and resistance *f*_D_, can be expressed by Eq. (32):

32 $$a_{0} = \frac{{F_{0} - \frac{1}{2}C_{D} \rho (t)Av_{0}^{2} - C_{M} M_{0} \frac{dv}{{dt}}}}{{m_{0} }}$$

By substituting Eq. (33) into (31), the tooth movement prediction model can be obtained. The following equation can be obtained:

33 $$S_{m} = v_{0} t_{1} + \frac{{F_{0} - \frac{1}{2}C_{D} \rho (t)Av_{0}^{2} - C_{M} M_{0} \frac{dv}{{dt}}}}{{2m_{0} }}t_{1}^{2}$$

where *S*_m_ is the amount of tooth movement under the action of the T-loop in the waxy model, *m*_0_ is the mass of the tooth, and *t*_1_ is the time of tooth movement.

### Tooth movement FEM analysis

**Fig. 4:**
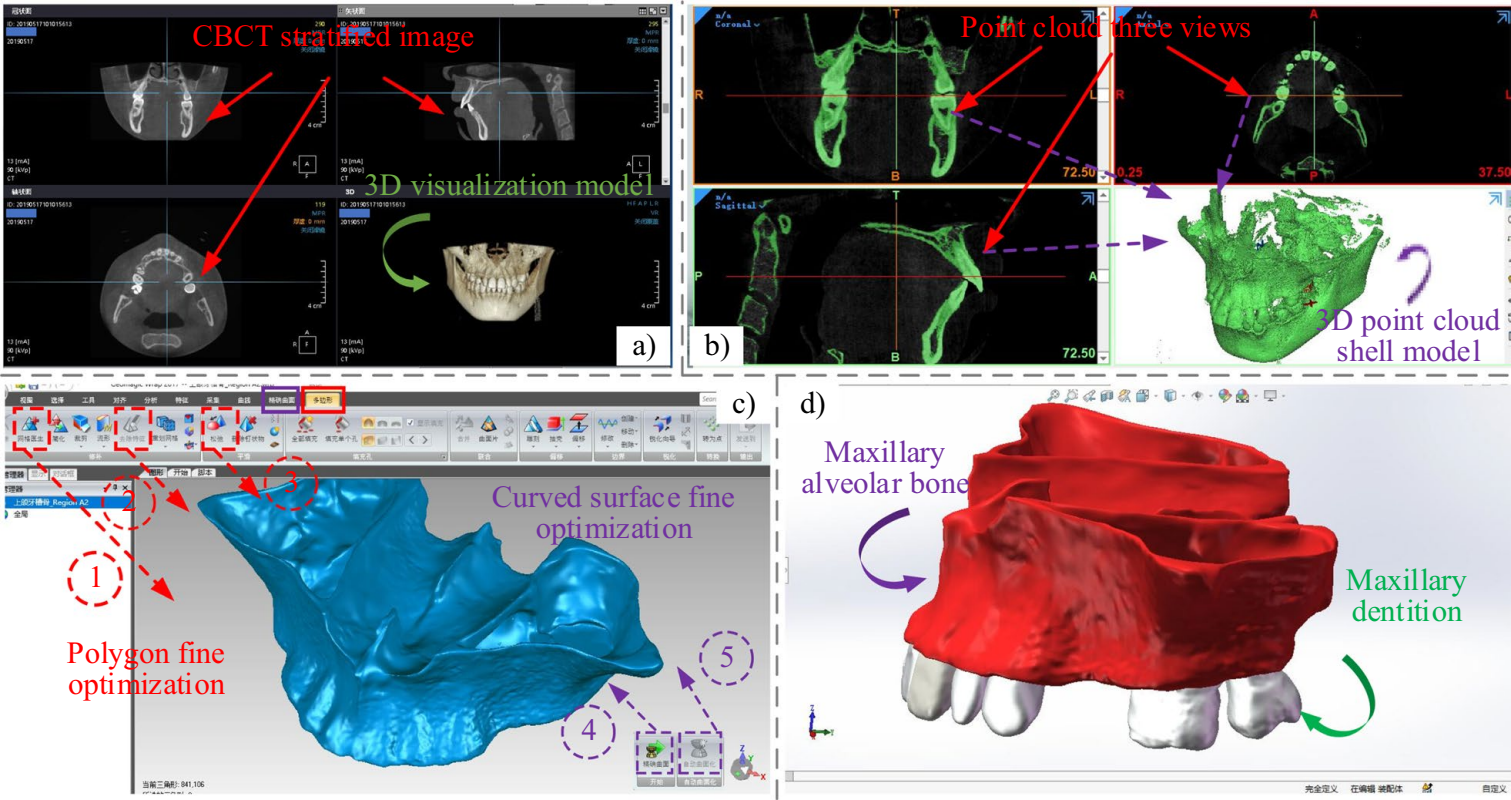
Flowchart of reverse 3D reconstruction of maxilla and dentition. (**a**) CBCT images; (**b**) one and three dimensional point cloud images in Mimics; (**c**) point cloud model surfacing and model optimization in Geomagic; (**d**) assembly of reconstructed models of the maxillary parts in SolidWorks

**Fig. 5:**
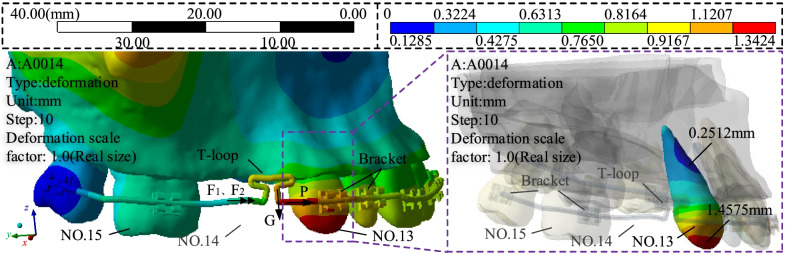
T-loop based maxillary biomechanical FEM analysis of the deformation cloud

In this study, a 3D model of the complete maxillary bone, dental row and periodontal ligament is reconstructed in reverse by using CBCT images (EWOO-VATECH Co., Ltd., Korea, scan thickness 0.5 mm, 301 scanned images) based on the patient's skull. And according to the clinical orthodontic treatment process, a 3D model with brackets and T-loop is installed on the reconstructed dental. At last, the orthodontic biomechanical FEM analysis is performed for the complete assembly model. The flowchart of the reverse 3D reconstruction model is shown in Fig. 4. And the simulation cloud is shown in Fig. 5. In the simulation process, the archwire is adopted of stainless steel archwire and Australian archwire, which are commonly used in clinical orthodontics. In addition, the material properties of the brackets and maxillary components are shown in Table 1 [34, 35, 36].

**Table 1:** Properties of the materials used in the FEM analysis

Material Properties	Cortical bone	Cancellous bone	Periodontal ligament	Tooth
Poisson's ratio ν	0.30	0.30	0.45	0.30
Young's modulus E(GPa)	13.00	0.345	0.00689	20.20
Density (mg/cm^3^)	2400	1100	–	–

	Brackets	Stainless steel archwire	Australian archwire
Poisson's ratio ν	0.30	0.30	0.30
Young's modulus E (GPa)	19.30	85.20	74.90
Elastic limit σ_e_ (GPa)	2.05	2.06	2.08
Tensile strength σ_b_ (GPa)	1.04	2.12	2.30

The simulation parameters shown in Fig. 5 are: (1) The archwire is a 0.014-inch round Australian archwire. (2) The material of the bracket is stainless steel. (3) The top of the maxilla is fixedly constrained. (4) The contact between the archwire and the brackets, between the brackets and the teeth is bound. (5) The archwire is meshed with 0.1 mm size and the others are meshed with 1.0 mm, so that the deformation of the archwire can be calculated more accurately. And the total number of nodes and cells of the mesh is 235,827 and 138,031 respectively. In addition, the displacement load is defined as follows: set 10 analysis steps so that the clearance distance grows linearly with 0.1 of the step length, and take the clearance distance as 0.3–1.2 mm for FEM analysis. And the maximum displacement of NO. 13 tooth is 1.4575 mm at the cusp and the minimum displacement is 0.2512 mm at the root, which meets the clinical orthodontic requirements [37, 38].

### Tooth movement experimental measurement method

**Fig. 6:**
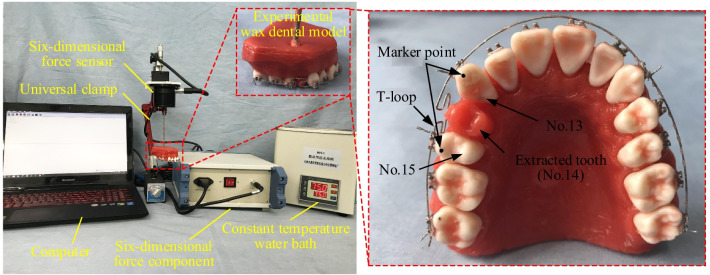
Installed waxy dental model with T-loop and tooth movement experiment system

In this study, the waxy model is used to simulate the periodontal tissue structure. Firstly, alginate is used as the impression material, and the separated teeth are placed into the alginate negative mold, then the molten waxy base is poured, left to cool and removed. Thus, the preparation of waxy dental model for experimental use is completed. Due to the viscoelastic properties at the corresponding temperature, the waxy model under the action of external force will produce a corresponding elastic deformation [28]. Finally, the teeth on the waxy model will complete the simulation of the orthodontic process with the change of time. The experimental system composition and the waxy dental model are shown in Fig. 6. Also detailed waxy model preparation process and modeling methods have been described in the study of Huang et al. [32].

The position of each maxillary teeth is marked using the FDI (Fédération Dentaire Internationale) marking method, and maxillary teeth No. 13 and No. 15 are selected as the teeth for the experimental study. Before clinical orthodontic treatment, a total of four teeth in the maxilla and mandible are usually extracted to allow enough space for remaining teeth to move. In the experiment, tooth No.14 is extracted and the two horizontal arms of the T-loop are ligated into the brackets of teeth No. 13 and No. 15. The highest points of the marginal crests of the two teeth are marked, as shown in Fig. 6. The distance between the two marker points is measured before the water-bath experiment, and then the waxy model is immersed in a constant temperature water-bath at 75 °C for 2 min. After taking out, the distance between the two marker points is measured again with a vernier caliper [32]. The difference of the distance between the two marker points before and after the water-bath is sought as the experimental value of the tooth movement distance under the action of T-loop.

## Results

**Fig. 7:**
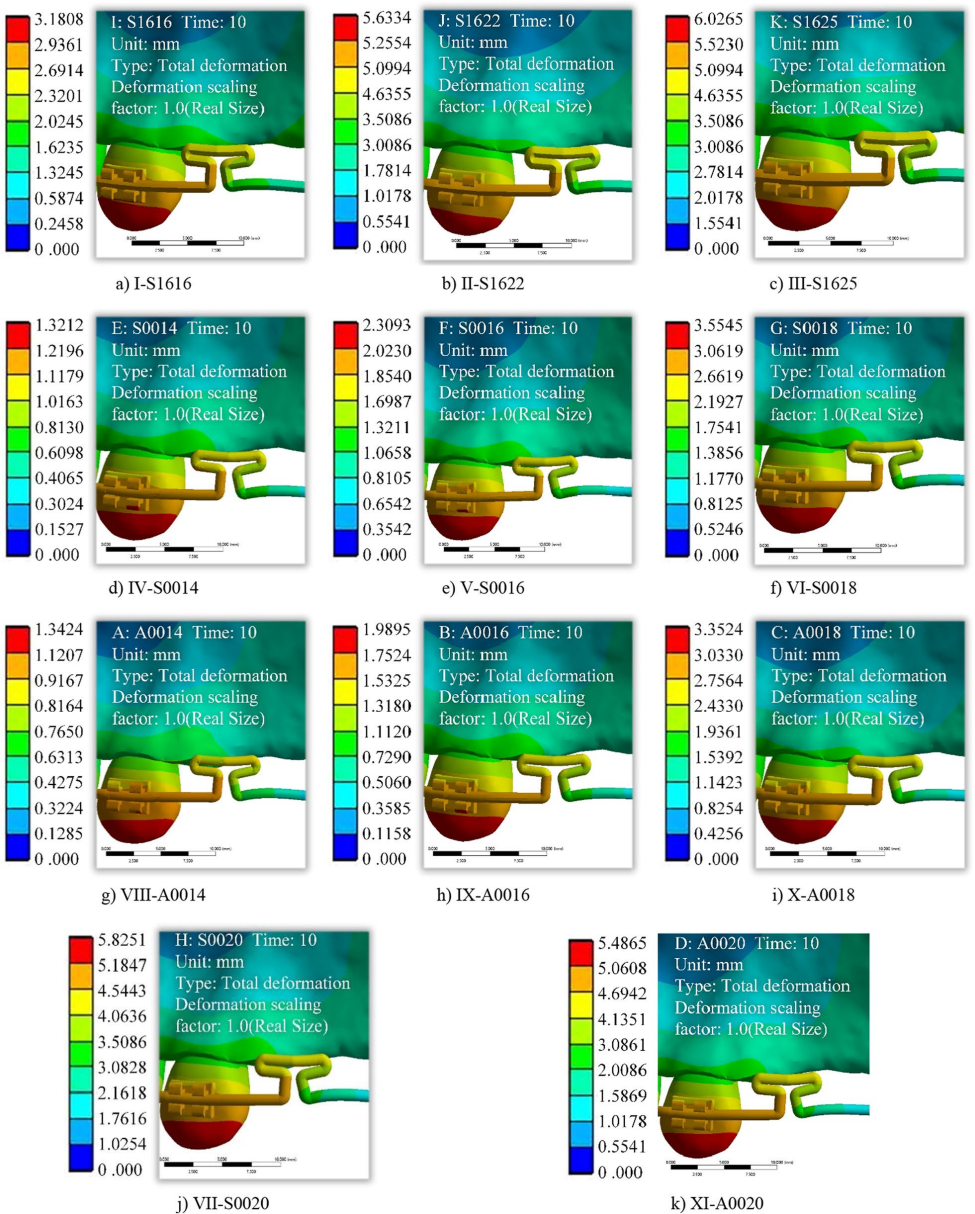
Total strain clouds for biomechanical FEM analysis of tooth movement based on T-loop

In clinical treatment, the magnitude of orthodontic force is changed by adjusting the characteristic parameters of the T-loop. And the magnitude of orthodontic force has an important influence on the tooth movement, the periodontium disease, tooth root resorption, and the alveolar bone reconstruction [39]. Therefore, in this study, the accuracy and reliability of the tooth movement prediction model is verified by comparing and analyzing the calculated, simulation and experimental data. In the FEM analysis, 11 kinds of T-loop 3D models with different parameters are established based on clinical orthodontic treatment. The loading process of the FEM analysis is the same as described before, with the aim of investigating the effects of different clearance distances, cross-sectional and materials on tooth movement. To be easily represented, the orthodontic archwires are codenamed as follows: I-S1616, II-S1622, III-S1625, IV-S0014, V-S0016, VI-S0018, VII-S0020, VIII-A0014, IX-A0016, X-A0018, XI-A0020. Where S is stainless steel archwire, A is Australian archwire, 1616 indicates 0.016 × 0.016 inch rectangular archwire, and 0014 indicates 0.014 inch round archwire. Similarly, the 11 kinds of T-loop bending parameters used in the experimental are the same as those of the 3D model, and 10 measurement points are set at 0.1 mm intervals to collect data during the loading measurement stage of the experiment. The calculated, simulation and experimental data are shown in Table 2. As well, the total strain clouds for the biomechanical FEM analysis of tooth movement under the action of 11 kinds of T-loop are shown in Fig. 7.

**Table 2:** Summary of calculated, simulation and experimental data

Clearance distance (mm)	Archwire codename										
	I	II	III	IV	V	VI	VII	VIII	IX	X	XI
*Calculated data (mm)*											
0.30	1.03	1.44	1.64	0.36	0.60	0.99	1.51	0.32	0.54	0.87	1.32
0.40	1.41	1.92	2.18	0.48	0.81	1.32	2.01	0.42	0.72	1.16	1.76
0.50	1.735	2.4	2.725	0.6	1.03	1.65	2.51	0.525	0.9	1.445	2.2
0.60	2.06	2.88	3.27	0.72	1.25	1.98	3.01	0.63	1.08	1.73	2.64
0.70	2.44	3.36	3.82	0.84	1.46	2.31	3.51	0.74	1.26	2.02	3.08
0.80	2.82	3.84	4.37	0.96	1.63	2.63	4.02	0.85	1.44	2.31	3.52
0.90	3.14	4.32	4.91	1.08	1.84	2.96	4.52	0.95	1.62	2.60	3.96
1.00	3.47	4.80	5.46	1.20	2.06	3.28	5.02	1.06	1.80	2.89	4.41
1.10	3.84	5.28	6.00	1.32	2.29	3.62	5.52	1.16	1.98	3.18	4.85
1.20	4.16	5.76	6.55	1.44	2.50	3.95	6.02	1.27	2.17	3.47	5.29
*Simulation data (mm)*											
0.30	0.98	1.73	1.85	0.41	0.71	1.08	1.76	0.41	0.61	0.99	1.69
0.40	1.22	2.17	2.32	0.51	0.89	1.35	2.20	0.51	0.76	1.24	2.11
0.50	1.47	2.60	2.78	0.61	1.07	1.62	2.64	0.61	0.91	1.49	2.53
0.60	1.71	3.03	3.25	0.71	1.24	1.88	3.08	0.71	1.06	1.74	2.95
0.70	1.96	3.47	3.71	0.81	1.42	2.15	3.52	0.82	1.21	1.99	3.37
0.80	2.20	3.90	4.17	0.91	1.60	2.42	3.96	0.92	1.36	2.24	3.80
0.90	2.45	4.33	4.64	1.02	1.78	2.69	4.40	1.02	1.52	2.48	4.22
1.00	2.69	4.77	5.10	1.12	1.95	2.96	4.84	1.12	1.67	2.73	4.64
1.10	2.94	5.20	5.56	1.22	2.13	3.23	5.28	1.22	1.82	2.98	5.06
1.20	3.18	5.63	6.03	1.32	2.31	3.50	5.73	1.32	1.97	3.23	5.48
*Experimental data (mm)*											
0.30	0.96	1.32	1.51	0.33	0.54	0.91	1.44	0.29	0.49	0.79	1.22
0.40	1.32	1.86	2.07	0.45	0.75	1.19	1.89	0.39	0.66	1.08	1.62
0.50	1.59	2.29	2.54	0.64	0.94	1.51	2.31	0.48	0.94	1.39	2.08
0.60	1.89	2.65	2.98	0.66	1.14	1.79	2.82	0.61	0.99	1.65	2.46
0.70	2.24	3.24	3.65	0.79	1.34	2.24	3.38	0.69	1.18	2.11	2.89
0.80	2.65	3.64	4.16	0.89	1.51	2.51	4.16	0.81	1.34	2.23	3.34
0.90	2.92	4.19	4.76	1.18	1.93	3.11	4.38	0.89	1.48	2.48	3.79
1.00	3.34	4.54	5.26	1.09	2.13	3.19	4.87	0.98	1.67	2.72	4.26
1.10	3.54	4.89	5.87	1.24	2.19	3.47	5.39	1.07	1.83	3.09	4.97
1.20	3.87	5.56	6.31	1.31	2.34	3.74	5.86	1.18	1.98	3.34	5.11

In this paper, the simulation data and experimental data are compared and analyzed, so that the accuracy and reliability of simulation analysis and experimental measurement can be verified with each other.

**Fig. 8:**
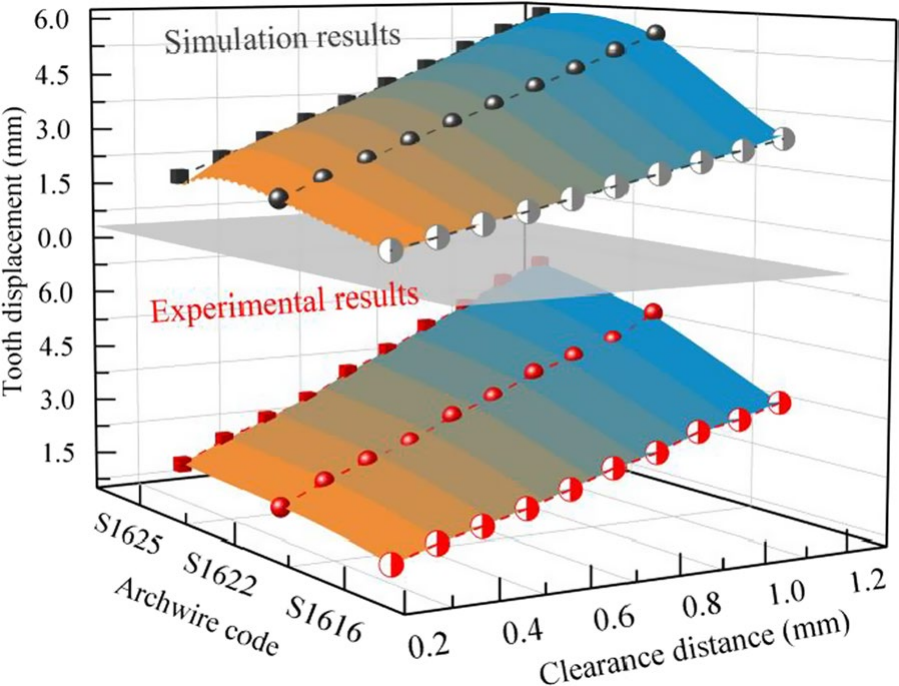
Tooth displacement produced by rectangular stainless steel archwire with different cross-sectional widths and clearance distances

Figure 8 shows the effect of rectangular archwire with different cross-sectional widths (Codenamed as I, II, and III) on the variation of tooth displacement with T-loop clearance distance. Both simulation and experimental results shows that the tooth displacement is positively correlated with the cross-sectional width for the same clearance distance. Similarly, the tooth displacement is positively correlated with the clearance distance for the same cross-sectional width. In addition, the effect of cross-sectional width on tooth displacement increases as the clearance distance increases.

**Fig. 9:**
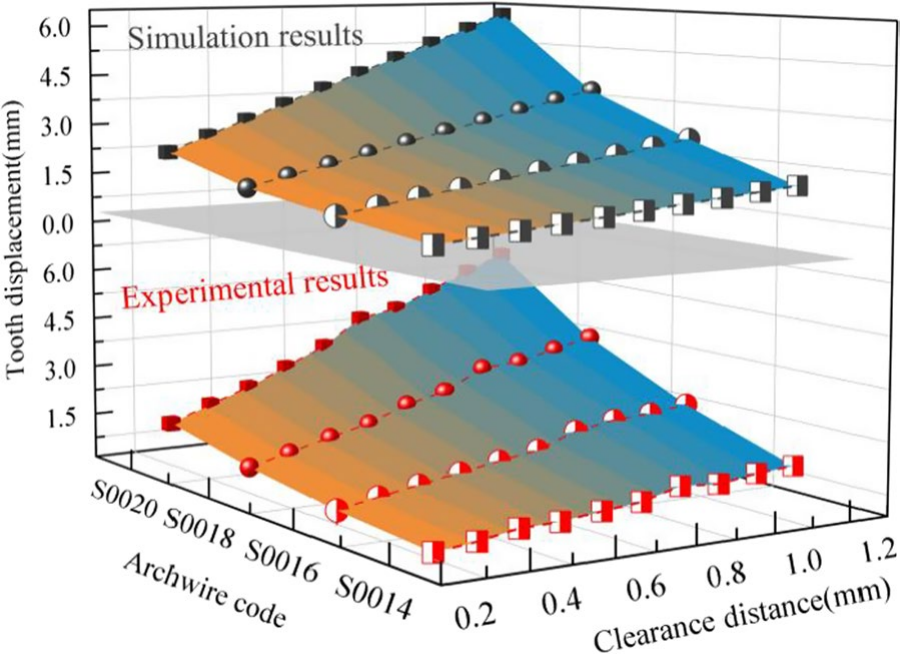
Tooth displacement produced by round stainless steel archwire with different cross-sectional diameters and clearance distances

Comparing different round cross-sectional diameter (Codenamed as IV, V, VI, and VII), the effect of tooth displacement with the variation of T-loop clearance distance is shown in Fig. 9. Both simulation and experimental results show that the tooth displacement is positively correlated with the cross-sectional diameter of the T-loop for the same clearance distance, which is similar to the conclusion for rectangular archwire. From the simulation and experimental results, although the cross-sectional diameter of the archwire affects the tooth displacement, its effect is weaker than that of the clearance distance of the T-loop.

**Fig. 10:**
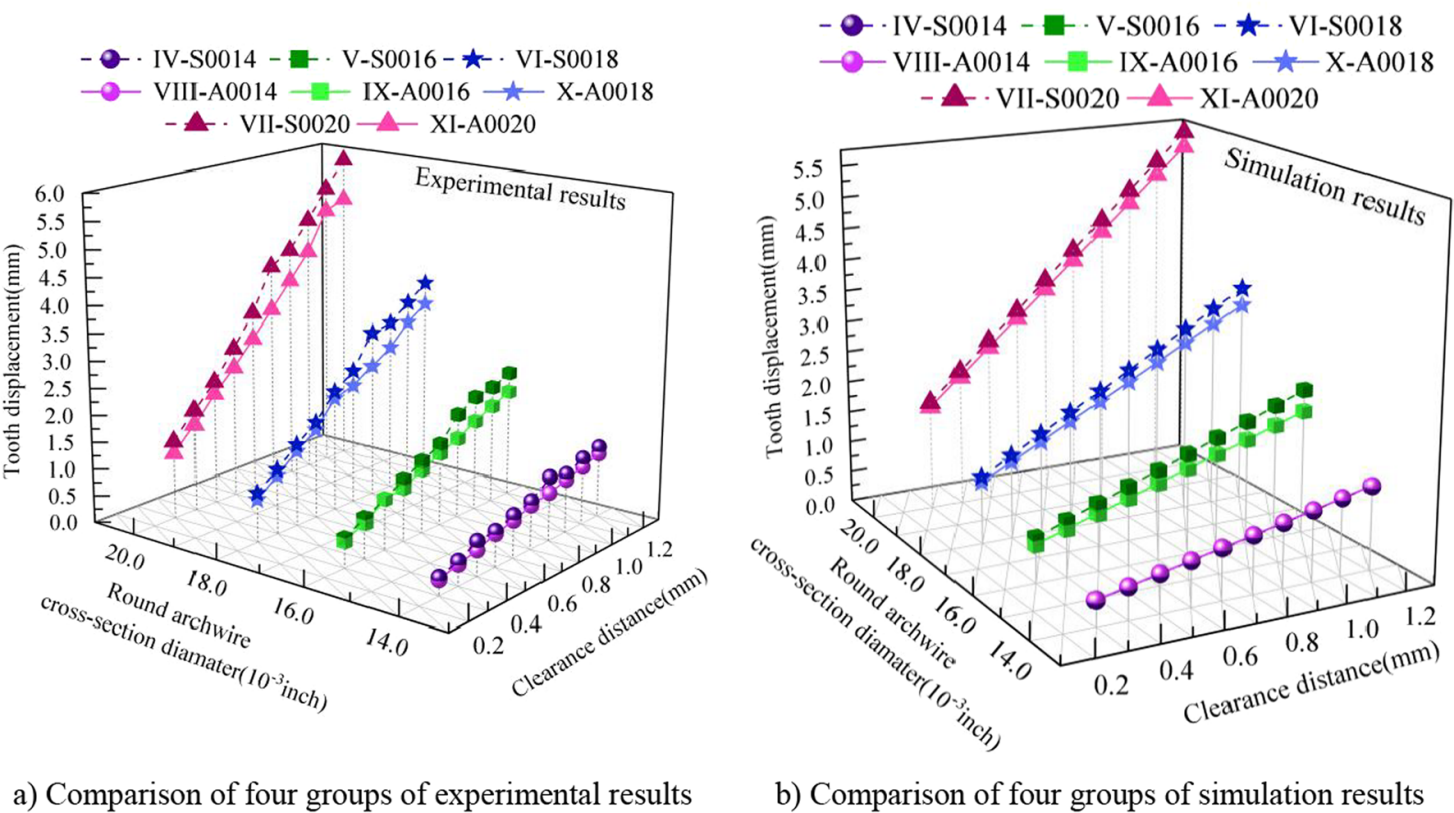
Comparison of tooth displacement with different archwire materials

In clinical orthodontic treatment, stainless steel archwire and Australian archwire are mainly used. Therefore, two sets of results based on material are divided. The data of four identical archwire types in the two sets are compared to investigate the effect of different archwire materials on tooth displacement [5, 32]. The stainless steel archwires are codenamed as IV, V, VI, and VII, and the Australian archwires are codenamed as VIII, IX, X, and XI. As shown in Fig. 10, both the simulation and experimental data show that the effect of tooth displacement with the same cross-sectional dimensions is more pronounced with increasing clearance distance using stainless steel archwire than Australian archwire. In addition, this conclusion became more evident as the diameter of the archwire increased.

**Fig. 11:**
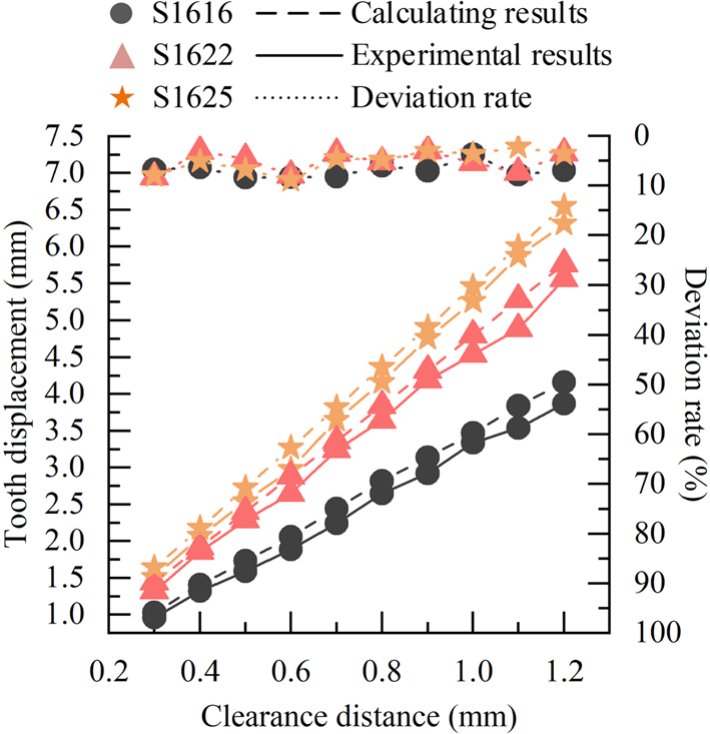
Comparison of experimental and calculated data with different cross-sectional widths, and deviation rate

**Fig. 12:**
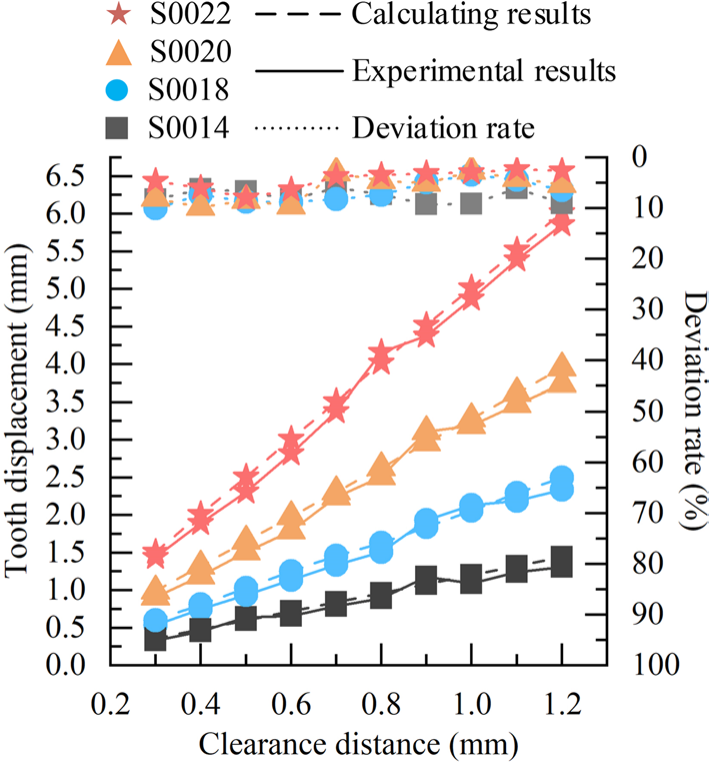
Comparison of experimental and calculated data of stainless steel archwire at different clearance distances, and deviation rate

**Fig. 13:**
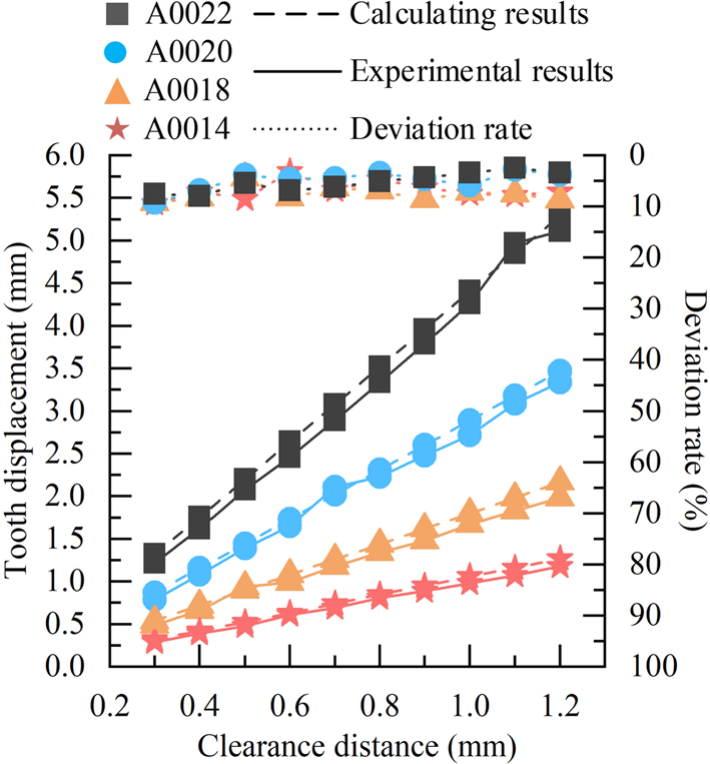
Comparison of experimental and calculated data of Australian archwire at different clearance distances, and deviation rate

While mutually verifying the accuracy and reliability of the simulation analysis and experimental measurements, this paper analyses the calculated and experimental data, and performs deviation calculations to verify the accuracy and validity of the tooth movement prediction model. The calculated data is shown in Table 2. And in Figs. 11, 12, 13, the experimental and calculated data, and deviation rates are shown by solid, dashed and dotted lines, respectively.

A comparison of experimental and calculated data of tooth displacement versus clearance distance for rectangular stainless steel archwire of different cross-sectional widths (Codenamed as I, II and III) is shown in Fig. 11. The obtained calculated and experimental data have the same trend and the deviation varies from 2.17  to  8.86%. When other conditions are the same, the trend of the curves shows that the calculated data of tooth displacement are positively correlated with the cross-sectional width, and the effect of the cross-sectional width on tooth displacement increases with the increase of the clearance distance.

For the round archwire, the comparison of experimental and calculated data of tooth displacement for different cross-sectional sizes of stainless steel archwire (Codenmed as IV, V, VI, VII) and Australian archwire (codenamed as VIII, IX, X, XI) at different clearance distances, and deviation rates are shown in Figs. 12 and 13. The trends of the calculated and experimental data are consistent in different archwire materials. The deviation rate of calculated and experimental data for stainless steel archwire ranges from 2.36  to  10.00%, while the deviation rate of Australian archwire ranges from 2.47  to  9.38%. For the two kinds of orthodontic archwire with different materials, the larger the cross-sectional size of the archwire, the greater the tooth displacement effect produces when the clearance distance is the same. When the cross-sectional size are all the same, the tooth displacement effect produced by the round orthodontic archwire is positively correlated with the clearance distance.

## Discussion

In order to reveal the tooth movement characteristics under different force during clinical orthodontic procedure, researchers have studied it through experimental measurements, FEM analysis and theoretical derivation. For example, researchers have established FEMs of tooth movement under different orthodontic force [40, 41, 42, 43]. During the simulation, the magnitude and direction of the orthodontic force is artificially set without reference, which is not consistent with the clinical reality. In addition, periodontal tissues cannot be effectively differentiated by materials, so corresponding experimental validation should be performed. However, experimental materials are mostly rigid models, such as plaster models or metal models [44], which lack biological properties. In addition, the orthodontic process is a dynamic one, and the orthodontic force will become weaker with the reduction of archwire deformation. Therefore, the real orthodontic effect cannot be effectively reproduced and the prediction function is not yet perfect. In contrast, the T-loop is a common orthodontic arch for clinical treatment of such oral malocclusion as closing the gap between teeth. Although the relationship between archwire shape and orthodontic force for T-loop has been studied to some extent [45, 46, 47, 48], but the archwire bending parameter and orthodontic force relationship, orthodontic force and tooth movement relationship have not been established. Therefore, the current studies still cannot assist dentists to effectively develop individualized T-loop parameter designs for different patients, as well as to accurately predict orthodontic treatment effect.

Thus, this paper firstly establishes a tooth movement prediction model based on the T-loop analysis and the waxy model dynamic resistance. Subsequently, to verify the accuracy of the prediction model, a reverse reconstruction model of the maxillary and dentition based on the patient's CBCT images is used to achieve a biomechanical FEM analysis of the tooth movement. Finally, a waxy dental model is established, and a water-bath measurement experiment mimicking the biological environment of the oral cavity is realized. Simulation analysis and experimental measurements are compared to verify each other's accuracy and reliability. And as shown in Figs. 8 and 9, the conclusion about the influence of different archwire parameters on tooth movement derived from the simulation analysis and experimental measurements is consistent with the established tooth movement prediction model. This further illustrates the accuracy of the simulation analysis and experimental measurements.

Afterwards, the prediction model and experimental measurements are compared and analyzed, and the deviation is calculated, and the total deviation range between the calculated and experimental data is obtained: 2.17  ~ 10.00%, which is within the acceptable range and is approved by the dentists of Peking University School of Dentistry. In addition, it can be determined from the trends shown in Figs. 11, 12 and 13 that the movement of the target tooth is positively correlated with both square and round archwire in terms of clearance distance and cross-sectional parameters. And the tooth displacement in the stainless steel archwire move faster than those in the Australian archwire as the clearance distance increases. The tooth displacement of the Australian archwire is milder for the same T-loop parameters. These trends and correlations are corrected with the existing academic studies in this field.

## Conclusions

In this paper, based on the analysis of the T-loop structure and the waxy model dynamic resistance, a tooth movement prediction model is established. The relationship between T-loop parameters, orthodontic force and tooth movement are revealed and quantified. The validity and reliability of the simulation analysis and experimental measurements, as well as the accuracy of the tooth movement prediction model, are verified by comparing and analyzing the simulation analysis and experimental measurements with each other, as well as calculating and analyzing the deviation of the experimental and calculated data.

Therefore, it can be affirmed that the established tooth movement prediction model has a high degree of confidence and can assist dentists in selecting the appropriate archwire parameters when bending T-loop to obtain the desired tooth movement distance. At the same time, the tooth movement prediction model provides dentists with an effective theoretical guide to avoid inefficient bending of archwire and improve the treatment effect of malocclusion. Moreover, the process and results of the FEM analysis enable predictive orthodontic treatment effects, thus laying the foundation for the realization of digital orthodontic treatment.

In future work, other detailed factors that may affect tooth movement during orthodontic treatment will be further explored. Such as the relative static friction and relative sliding friction between the orthodontic archwire and brackets, and how to choose the right archwire parameters to achieve the best orthodontic effects and speed up the treatment process.

## Data Availability

All relevant data are within the present paper; however, the ethics committee strictly forbids sharing the CBCT files of patients and control subjects that they contain some identifying information, according to its regulations on the data access. Therefore, CBCT data of the samples will not be shared. Also the reverse reconstructed 3D models in this paper are belong to the authors and are therefore available only upon request, after approval by all the authors. And if required, please contact the corresponding author of this paper (Correspondence: jiangjingang@hrbust.edu.cn).

## Abbreviations

FEM: Finite element method
3D: Three dimensional
FDI: Fédération dentaire internationale
